# Preparation and Performance Evaluation of High-Temperature Polymer Nano-Plugging Agents for Water-Based Drilling Fluids Systems Applicable to Unconventional Reservoirs

**DOI:** 10.3390/polym17050588

**Published:** 2025-02-23

**Authors:** Lei Yao, Xiaohu Quan, Yongjie Zhang, Shengming Huang, Qi Feng, Xin Zhang

**Affiliations:** 1 China Oilfield Services Ltd., Tianjin 300451, China; 17854227170@163.com (L.Y.); 19141943806@163.com (Y.Z.); 2 Oil and Gas Resources Research Center, Ningxia University, Yinchuan 750021, China; 3 Ministry of Education (MOE) Key Laboratory of Petroleum Engineering, College of Petroleum Engineering, China University of Petroleum (Beijing), Beijing 102249, China; smhuang2015@163.com (S.H.); 18563073299@163.com (Q.F.); 15020050834@163.com (X.Z.)

**Keywords:** high-temperature resistance, nanoparticles, nano-plugging agent, water-based drilling fluids gel system, unconventional reservoirs

## Abstract

To address the challenges of micro-fracture development in shale formations, frequent wellbore instability, and the limited plugging capability of water-based drilling fluids in unconventional reservoirs, a nano-plugging agent (NPA) was synthesized using emulsion polymerization. The synthesized NPA was characterized through thermogravimetric analysis (TGA) and transmission electron microscopy (TEM), revealing excellent high-temperature stability and a spherical or sub-spherical morphology, with particle diameters ranging from approximately 20 to 50 nm. The rheological, filtration, and plugging properties of NPA were systematically evaluated, and its sealing mechanism was analyzed. The results demonstrate that at a test temperature of 180 °C, the optimal NPA concentration in the drilling fluid base slurry is 1.5%, achieving a 60.5% reduction in HTHP (high-temperature high-pressure) sand disc filtration loss. Additionally, the API filtration loss and HTHP filtration loss reduction rates reached 58.1% and 50.3%, respectively, highlighting the remarkable filtration loss reduction and plugging efficiency of NPA under high-temperature conditions. After NPA treatment, the specific surface area and pore volume of shale cuttings decreased to 9.348 m^2^/g and 0.035 cm^3^/g, respectively, indicating effective surface plugging. The mechanism analysis suggests that due to its nanoscale size, NPA can penetrate deep into micro-pores and fractures within the shale, achieving deep-layer plugging. Furthermore, NPA forms a physical plugging barrier on the shale surface, effectively suppressing shale hydration and swelling. This study provides valuable insights and guidance for addressing wellbore instability and the insufficient plugging performance of drilling fluids in unconventional reservoir drilling operations.

## 1. Introduction

Unconventional reservoirs, such as shale oil and gas, have emerged as a vital component of the global energy supply, largely due to advancements in horizontal drilling and hydraulic fracturing technologies [1,2,3]. However, the unique geological and petrophysical properties of these reservoirs—characterized by ultra-low permeability, complex pore structures, and the presence of micro-fractures—present significant challenges to drilling operations [4,5]. Among these, maintaining the stability of wellbores in shale formations stands out as one of the most critical issues. Shale formations are highly water-sensitive, and exposure to water-based drilling fluids often results in hydration and swelling. This process exacerbates the propagation of micro-fractures, disrupts pore pressure equilibrium, and can ultimately lead to wellbore collapse [6,7,8,9]. While oil-based or synthetic-based drilling fluids offer better performance in mitigating these issues, water-based drilling fluids (WBDFs) are preferred in many drilling operations due to their environmental compatibility, cost-effectiveness, and ease of handling [10,11,12,13,14,15]. Despite their advantages, WBDFs face significant limitations in controlling fluid invasion and stabilizing water-sensitive shale formations [16,17]. Traditional fluid loss additives primarily consist of larger particles that are incapable of effectively sealing the nanopores and micro-fractures prevalent in unconventional shale reservoirs. This shortcoming increases the risk of fluid invasion, intensifies hydration effects, and compromises the mechanical stability of the formation [18]. Consequently, there is an urgent need to develop advanced additives capable of addressing the dual challenges of fluid loss and wellbore instability, particularly under high-temperature and high-pressure (HTHP) conditions. Such innovations would represent a significant advancement in overcoming the limitations of existing water-based drilling fluids and ensuring safe, efficient drilling operations in unconventional reservoirs.

Nanotechnology provides a promising solution to overcome the limitations of traditional drilling fluid additives [19,20,21,22]. With their small size, high specific surface area, and tunable physicochemical properties, nanoparticles have shown significant potential as effective plugging agents in drilling fluids [23,24]. These nano-plugging agents can penetrate nanopores and micro-fractures, offering a robust sealing mechanism that surpasses the capabilities of conventional additives. Moreover, nanoparticles form a compact physical barrier on the shale surface, effectively preventing water intrusion, mitigating shale hydration and swelling, and significantly improving wellbore stability [25,26,27,28]. Research and practical applications have demonstrated that incorporating nano-plugging agents into water-based drilling fluids (WBDFs) enhances their plugging performance and wellbore stabilization capabilities. However, despite the growing adoption of nanotechnology in drilling operations, several critical scientific challenges persist [29]. The foremost challenge is the synthesis of nanomaterials with high thermal stability to ensure consistent performance under HTHP conditions. Shale reservoirs, often located at substantial depths, are subject to extreme temperature and pressure environments. To function effectively in these conditions, nano-plugging agents must not only exhibit excellent thermal stability but also retain their particle structure and functional properties under high-pressure conditions [30,31,32]. The second major challenge is ensuring the compatibility of nano-plugging agents with WBDFs components. The chemical environment of WBDFs is complex, consisting of clays, polymers, and various chemical additives. Under these conditions, nanoparticles may aggregate or settle, significantly reducing their plugging performance [33,34]. To address this, optimizing the surface modification and dispersion properties of nanoparticles is essential to enhance their stability and effectiveness in WBDFs. Research has shown that the concentration of nano-plugging agents directly influences the rheological properties and plugging performance of WBDFs. A concentration that is too low may fail to create an effective sealing barrier, while excessive concentrations could increase the viscosity of the drilling fluid, reducing its flowability and impairing drilling efficiency [35,36,37]. To address this, experimental and simulation studies are needed to identify the ideal dosage of nano-plugging agents for various downhole conditions. Huang et al. [38] synthesized a core-shell structured nanocomposite material designed for WBDFs. This material significantly improved plugging efficiency during shale gas drilling, enhanced wellbore stability, and reduced drilling fluid invasion into formations. Similarly, Jing et al. [39] developed a nano-plugging agent, AMPS/AM, that effectively sealed shale pore structures and improved wellbore stability during fracturing operations. Field tests in the Zhaotong block demonstrated its successful application and favorable results. Liu et al. [40] prepared a self-degrading shielding temporary plugging agent (DSTPA) using inverse emulsion polymerization. Studies revealed that DSTPA exhibited excellent pressure-bearing and plugging capabilities, achieving a plugging efficiency exceeding 90%. At a concentration of 1%, the system’s fluid loss was reduced by 24 mL. Additionally, DSTPA showed good high-temperature stability and degraded into a water-like solution, making it environmentally friendly and efficient. The application of nano-plugging agents in drilling for unconventional reservoirs represents a breakthrough in addressing the limitations of traditional WBDFs. By conducting in-depth studies on the mechanisms of action and modification methods of nanoparticles, their performance in plugging and wellbore stabilization under HTHP conditions can be further optimized. These advancements offer robust technical support for the efficient and sustainable development of unconventional oil and gas resources. Despite the advancements in nano-plugging agents for drilling fluids, several limitations still exist that hinder their widespread application in unconventional reservoirs. Current nano-sealing technologies face challenges such as inadequate thermal stability under high-temperature and high-pressure (HTHP) conditions, suboptimal dispersion in drilling fluids, and inconsistent sealing performance due to variations in shale microstructures. Additionally, some conventional nanoparticles tend to agglomerate, reducing their effectiveness in forming a uniform sealing barrier.

This study aims to address these issues through the development of a nano-plugging agent (NPA) with excellent thermal stability, improved dispersion properties, and enhanced plugging efficiency. We have developed a novel nano-plugging agent (NPA) using emulsion polymerization technology specifically designed to address the unique challenges posed by unconventional reservoirs. The nano-plugging agent NPA was characterized using thermogravimetric analysis (TGA) and transmission electron microscopy (TEM). TEM analysis confirmed that the synthesized NPA exhibited a spherical or sub-spherical morphology, with particle diameters ranging from 20 to 50 nm. To assess the performance of NPA, we conducted a comprehensive set of experiments, including sand disc plugging tests, specific surface area and pore volume analysis of shale samples, microscopic observation of shale morphology before and after plugging, high-temperature resistance tests, and comparative evaluations with commercial nano-plugging agents (NP-1 and NP-2). The high-temperature stability of NPA ensures its efficacy under HTHP conditions, and the synthesis method and components of NPA are optimized to make it suitable for harsh drilling environments. The proposed NPA not only achieves deep pore plugging, but also establishes a strong physical barrier on the shale surface, prevents the intrusion of leachate, and effectively inhibits well wall destabilization caused by hydration. The results of this study provide valuable insights into the design and application of nano plugging agents in WBDFs and offer a viable solution to the long-term challenges of wellbore instability and fluid loss in unconventional reservoirs.

## 2. Experimental Section

### 2.1. Materials and Instruments

Experimental Materials: The experimental materials included styrene (St), 2-acrylamido-2-methylpropanesulfonic acid (AMPS), potassium persulfate (KPS), acrylic acid (AA), dimethyldiallylammonium chloride (DMDAAC), and silane coupling agent (KH570), all sourced from Shanghai Aladdin Biochemical Technology Co., Ltd. (Shanghai, China). Span80, anhydrous sodium carbonate (Na_2_CO_3_), and anhydrous ethanol were also obtained from the same supplier. Silicon dioxide nanoparticles (30 ± 5 nm) were purchased from Jiangsu Xianfeng Nanomaterials Technology Co., Ltd. (Nanjing, China). Bentonite was procured from Shandong Huawei Bentonite Co., Ltd. (Weifang, China), while quartz sand (40–60 mesh, 60–80 mesh) was supplied by Energy Chemical Reagent Co., Ltd. (Shanghai, China). Deionized water used throughout the experiments was produced in the laboratory. The related materials and functions are shown in Table 1.

Experimental Instruments: The instruments used in this study included a BS224S electronic balance from Sartorius Scientific Instruments Co., Ltd. (Beijing, China), and a DF heat collection magnetic stirrer from Changzhou Danrui Instruments (Changzhou, China). A GZX constant temperature drying oven was supplied by Qingdao Lantun Instruments Co., Ltd. (Qingdao, China), and a ZNS-2 drilling fluid filtration apparatus and GJSS-B12K variable frequency high-speed stirrer were provided by Qingdao Haitongda Specialized Instrument Factory (Qingdao, China). Additional equipment included a BGRL-5 roller heating furnace, a GGS71 high-temperature high-pressure filtration loss apparatus, and a PPA drilling fluid plugging performance evaluation instrument, all from Qingdao Tongchun Instrument Factory (Qingdao, China). Analytical instruments included an SU8010 scanning electron microscope from Hitachi Corporation (Tokyo, Japan) and an ASAP 2460 BET surface area and pore size distribution analyzer from Micromeritics Corporation, Norcross, GA, USA.

### 2.2. Preparation of Nano-Plugging Agent

**Figure 1:**
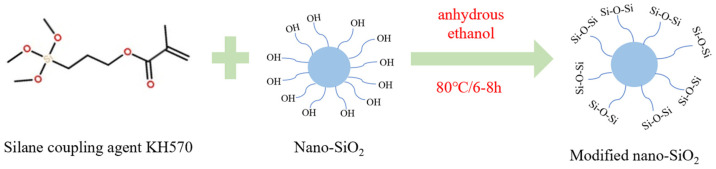
Schematic diagram of SiO_2_ nanomaterials modification.

First, as shown in Figure 1, 100 mL of anhydrous ethanol was placed in a beaker, followed by the addition of 0.25 wt% silica nanoparticles (30 ± 5 nm). The mixture was ultrasonically dispersed for 1–2 h. Meanwhile, 0.2 wt% of the silane coupling agent KH570 was dissolved in 50 mL of anhydrous ethanol and stirred thoroughly with a magnetic stirrer for 30 min. The two prepared solutions were then combined in a three-necked flask and heated to 80 °C for a high-temperature reaction lasting 6–8 h. After the reaction, the products were repeatedly washed with anhydrous ethanol and dried. Subsequently, 100 mL of deionized water and 2.5 wt% Span80 were added to a three-necked flask equipped with a condenser tube. After stirring, DMDAAC and the modified silica were introduced at a ratio of 0.5:0.25. Then, styrene, 2-acrylamide-2-methylpropanesulfonic acid, and acrylic acid monomers were sequentially added in a ratio of 1.2:0.8:1.5. The monomer mixture underwent ultrasonic dispersion for 1–2 h to ensure uniform distribution. Once fully dissolved and homogenized, the temperature was raised to 70 °C. Next, 0.1 wt% potassium persulfate (KPS) was added to initiate the polymerization reaction, which proceeded for 4 h. After completion, heating was stopped, and the resulting NPA was obtained by washing with anhydrous ethanol and drying for 24 h.

### 2.3. Characterization

#### 2.3.1. TGA Analysis

Using the TG209-F3 thermogravimetric analyzer, the Al_2_O_3_ crucible was placed in the sample chamber and calibrated to zero. Next, 0.4 g of the sample was accurately weighed and transferred into the Al_2_O_3_ crucible. The crucible, containing the sample, was then returned to the sample chamber, and the TGA program was initiated. The test involved heating the sample at a rate of 10 °C/min under a nitrogen purge atmosphere until a final temperature of 600 °C was reached. The thermogravimetric curve of the NPA sample was recorded and analyzed.

#### 2.3.2. TEM Analysis

Using the JEM-2100UHR transmission electron microscope (NEC, Tokyo, Japan), a small amount of the NPA sample was dispersed in ethanol and subjected to ultrasonic treatment to ensure uniform particle dispersion. A drop of the resulting dispersion was carefully placed onto a copper grid coated with either a carbon or silicate film. Once the solvent had fully evaporated, the sample was prepared for testing. The microscope’s acceleration voltage was set between 100 and 300 kV, depending on the specific requirements of the analysis.

### 2.4. Performance Methods

#### 2.4.1. Evaluation of Bentonite-Based Slurry Performance

To prepare a freshwater base slurry, 16 g of bentonite and 0.8 g of anhydrous sodium carbonate were added to 400 mL of deionized water and stirred at high speed for 20 min. The mixture was then sealed and allowed to cure at room temperature for 24 h. Next, varying amounts of the nano-plugging agent were incorporated into the base slurry to create drilling fluids with nano-plugging agent concentrations of 0 wt%, 0.5 wt%, 1.0 wt%, 1.5 wt%, and 2.0 wt%. After high-speed stirring for 20 min, the rheological and filtration properties of these drilling fluids were measured. The measurements were conducted both before and after 16 h of high-temperature aging at 150 °C, following the GB/T 16783-2014 standard [41]. Apparent viscosity (AV, mPa·s) and plastic viscosity (PV, mPa·s) were calculated by Equations (1) and (2).

(1) $$\mathrm{AV}=\varphi_{600}/2$$

(2) $$\mathrm{PV}=\varphi_{600}-\varphi_{300}$$

where *φ*_600_ and *φ*_300_ are the viscometer readings at 600 rpm and 300 rpm, respectively.

#### 2.4.2. Evaluation of Sand Disk Plugging Performance

The plugging performance of the nano-plugging agent was assessed using a PPA-type drilling fluid plugging performance evaluation instrument. The nano-plugging agent was added to the base slurry, and the mixture underwent high-temperature aging for 16 h at 150 °C and 180 °C. Following the aging process, the drilling fluids were tested under simulated high-temperature and high-pressure conditions using a heating jacket to replicate formation environments. To mimic underground formation pores, a ceramic sand disk with 5 μm fractures was employed. The high-temperature, high-pressure (3.5 MPa) sand disk filtration loss was then measured at both 150 °C and 180 °C.

#### 2.4.3. Specific Surface Area and Pore Volume Testing of Rock Samples

Nitrogen adsorption experiments provide an accurate method for analyzing the pore characteristics of rocks. Shale cuttings, sized between 6 and 10 mesh, were used to evaluate the plugging performance of the nano-plugging agent by examining changes in their specific surface area and pore volume before and after plugging. A solution containing a specific concentration of the nano-plugging agent was prepared using a vacuum filtration bottle. Approximately 10–20 g of shale cuttings were soaked in 350 mL of the high-temperature-resistant nano-plugging agent solution for 6–8 h under a vacuum pressure of 0.08 MPa. After soaking, the shale cuttings were dried for 5 h. Subsequently, nitrogen adsorption experiments were conducted using an ASAP 2460 BET surface area and pore size distribution analyzer to determine the specific surface area and pore volume of the shale samples.

#### 2.4.4. Evaluation of Micro-Morphology Before and After Shale Plugging

Shale cuttings, sized between 6 and 10 mesh, were soaked in a solution containing a specific concentration of high-temperature-resistant nano-plugging agent and aged at 150 °C for 16 h. Following the aging process, the cuttings were filtered using a 40-mesh sieve and subsequently dried for 5 h. The microscopic morphology of the shale cuttings after treatment with the nano-plugging agent was then examined using an SU8010 scanning electron microscope.

#### 2.4.5. Evaluation of Temperature-Resistant Stability Performance

A specific amount of nano-plugging agent was added to the drilling fluid base slurry and stirred vigorously for 20 min. The rheological and filtration properties of the drilling fluid were subsequently evaluated both before and after 16 h of high-temperature aging at 150 °C, 180 °C, and 200 °C, according to the standard of GB/T 16783-2014, “Field Testing of Drilling Fluids in the Oil and Natural Gas Industry, Part 1: Water-Based Drilling Fluids”.

#### 2.4.6. Comparative Evaluation of Different Plugging Agents

To validate the performance of the synthesized high-temperature-resistant nano-polymer plugging agent, a comparison was conducted with two commonly used nano-plugging agents, NP-1 and NP-2, utilized in oilfield operations. The rheological properties and filtration performance of the drilling fluids were evaluated both before and after 16 h of aging at 150 °C, 180 °C, and 200 °C. A 1.5% concentration of the high-temperature-resistant nano-plugging agent (NPA), NP-1, and NP-2 was added to the base drilling fluid slurry, and the mixtures were stirred vigorously for 30 min. The rheological and filtration properties were then measured after the high-temperature aging tests, in accordance with the GB/T 16783-2014 standard.

## 3. Results and Discussion

### 3.1. TGA Analysis

**Figure 2:**
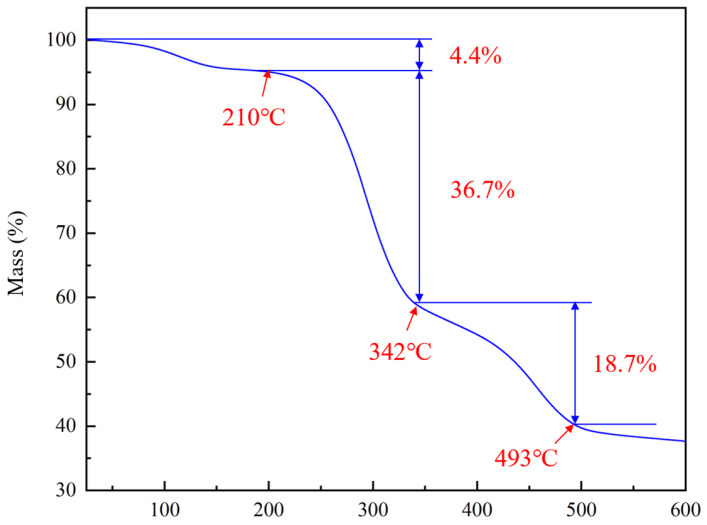
TGA curve of nano plugging agent NPA.

The thermogravimetric analysis (TGA) curve of the high-temperature-resistant nano-plugging agent is presented in Figure 2. From room temperature to approximately 210 °C, the nano-plugging agent exhibits a mass loss rate of 4.4%, likely attributed to the evaporation of free water present in the nano-plugging agent’s powder. Between 210 °C and 342 °C, the mass loss rate increases to 36.7%, which may result from the decomposition of minor molecular side groups, such as amide groups or benzene rings [42,43]. A significant mass loss occurs between 342 °C and 493 °C, with a loss rate of 18.7%, potentially due to the degradation of the polymer’s main chain. Beyond 493 °C, the molecular structure of the polymer undergoes complete thermal degradation. The presence of sulfonic acid groups, benzene rings, and other rigid structural units enhances the polymer’s chain rigidity, contributing to the nano-plugging agent’s improved high-temperature stability. Overall, the analysis indicates that the nano-plugging agent (NPA) demonstrates excellent thermal stability.

### 3.2. TEM Analysis

**Figure 3:**
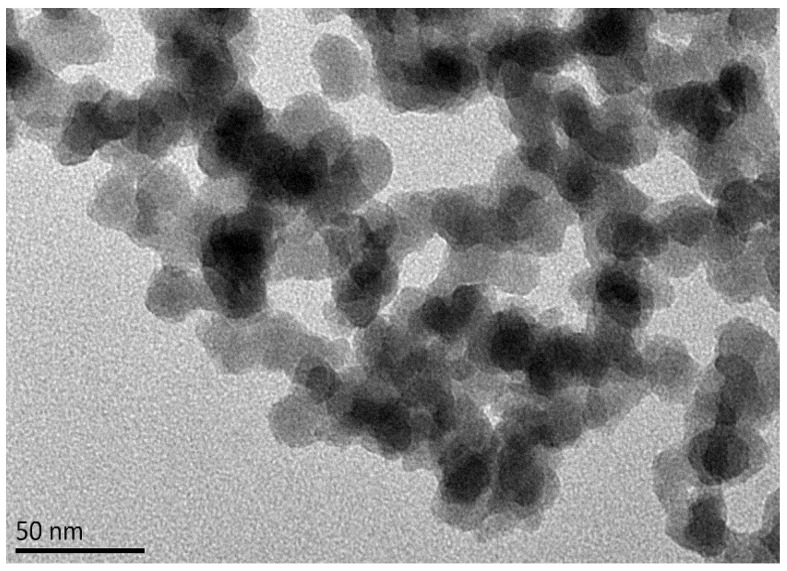
TEM image of nano plugging agent NPA.

The transmission electron microscope (TEM) image of the high-temperature-resistant nano-plugging agent is shown in Figure 3. TEM observations indicate that the nano-plugging agent (NPA) particles have a diameter ranging from approximately 20 to 50 nm and exhibit a relatively uniform spherical or near-spherical morphology. This suggests that the silica-modified particles are well-controlled in terms of their shape and size. While some particle aggregation is observed, the overall distribution remains relatively uniform, without any significant large-scale agglomeration. This uniform dispersion is likely attributed to the optimization of the modification process, which effectively enhanced the dispersion properties of the NPA particles.

### 3.3. Evaluation of Bentonite-Based Slurry Performance

**Figure 4:**
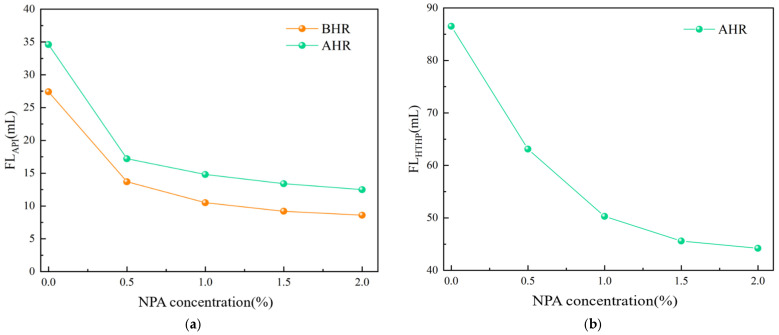
Effect of nano plugging agent NPA concentration on drilling fluid filtration performance. (**a**) Effect of NPA on API filtration loss before and after aging. (**b**) Effect of NPA on HTHP filtration loss after aging.

The changes in the rheological properties and filtrate loss of the drilling fluid with varying concentrations of the nano-plugging agent (NPA) added to the base slurry are presented in Table 2 and Figure 4, as evaluated using the method described in Section 2.4.1. According to Table 1, under room temperature conditions and after high-temperature aging at 150 °C, the addition of 0 wt%, 0.5 wt%, 1.0 wt%, 1.5 wt%, and 2.0 wt% NPA results in minimal changes in the Apparent Viscosity (AV) and Plastic Viscosity (PV) of the drilling fluid compared to the base slurry. This indicates that the NPA has a negligible effect on the rheological properties of the drilling fluid. Moreover, the addition of NPA effectively reduces the filtrate loss of the base slurry. As the concentration of NPA increases, the filtrate loss decreases. Notably, when the concentration is below 1.5 wt%, the reduction in filtrate loss is significant. However, beyond 1.5 wt%, although the filtrate loss continues to decrease, the reduction becomes less pronounced. Considering both drilling performance and cost-efficiency, the optimal NPA concentration is determined to be 1.5 wt%. From Figure 3, it can be observed that under room temperature conditions, adding 1.5 wt% NPA reduces the API filtrate loss of the base slurry from 27.4 mL to 9.2 mL, achieving a 66.4% reduction. After aging at 150 °C, the API filtrate loss is further reduced from 34.6 mL to 13.4 mL, with a reduction rate of 61.3%. The NPA also significantly decreases HTHP filtrate loss. After aging at 150 °C, the HTHP filtrate loss of the base slurry is reduced from 86.5 mL to 45.6 mL, representing a 47.3% reduction. These results demonstrate that the NPA effectively reduces both API and HTHP filtrate losses in drilling fluids and exhibits excellent thermal stability.

### 3.4. Evaluation of Sand Disk Plugging Performance

**Figure 5:**
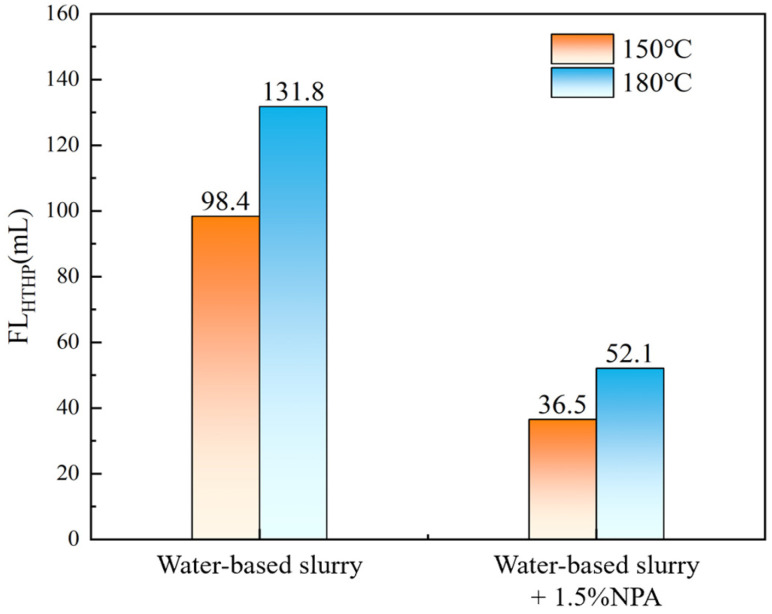
Evaluation results of plugging performance of nano plugging agent NPA sand disk.

The evaluation results of the sand screen plugging performance of the nano-plugging agent NPA, as shown in Figure 5 and based on the testing method outlined in Section 2.4.2, indicate its effectiveness under high-temperature conditions of 150 °C and 180 °C. The HTHP sand screen filtrate loss of the base slurry is measured at 98.4 mL and 131.8 mL, respectively. However, with the addition of 1.5% NPA, the filtrate loss decreases significantly to 36.5 mL and 52.1 mL, representing reductions of 62.9% and 60.5%, respectively. These results demonstrate that the nano-plugging agent NPA can effectively seal sand screen pores under high-temperature and high-pressure conditions, significantly reducing the filtrate loss of drilling fluid and exhibiting excellent plugging performance. This superior performance is attributed to the spherical, flexible particles of NPA, which can elastically deform to adapt to different pore openings. This adaptability allows the agent to effectively fit and fill micro-pores and cracks of various shapes. Additionally, NPA’s strong adsorption capability on rock surfaces further enhances its ability to reduce filtrate loss in drilling fluids.

### 3.5. Specific Surface Area and Pore Volume Testing of Rock Samples

Following the method outlined in Section 2.4.3, the specific surface area and pore volume of the original shale, as well as those of shale treated with deionized water and a nano-plugging agent solution, were tested. The experimental results are presented in Table 3. The original shale exhibited the smallest specific surface area and pore volume, measured at 6.721 m^2^/g and 0.016 cm^3^/g, respectively. After treatment with deionized water, the shale underwent hydration expansion, resulting in significant increases in both specific surface area and pore volume. The values for the treated shale were 16.416 m^2^/g and 0.016 cm^3^/g, respectively. In contrast, shale treated with the nano-plugging agent NPA showed a specific surface area of 9.348 m^2^/g and a pore volume of 0.035 cm^3^/g. Although these values were slightly higher than those of the original shale, they were significantly lower than those of the shale treated with deionized water. This demonstrates that the nano-plugging agent NPA effectively reduced the pore structure’s expansion by plugging the shale surface, thereby exhibiting a good plugging effect.

### 3.6. Evaluation of Micro-Morphology Before and After Shale Plugging

**Figure 6:**
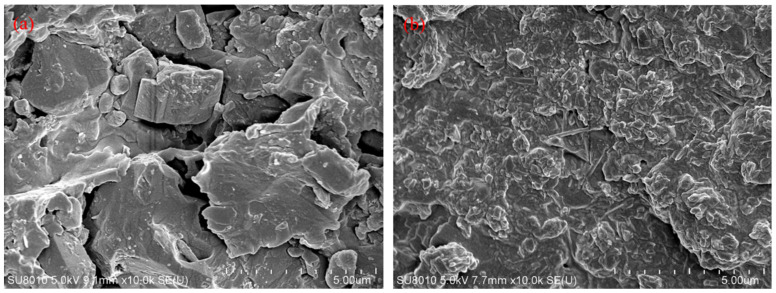
SEM image of micro-morphology before (**a**) and after (**b**) shale plugging.

Using the testing method outlined in Section 2.4.4, rock cuttings were soaked in a 1.5 wt% nano-plugging agent (NPA) solution. The scanning electron microscope (SEM) images of the shale surface before and after plugging are shown in Figure 6. Figure 6a illustrates that the original rock cuttings contain numerous pores and fractures, which facilitate the leakage of drilling fluid. In contrast, Figure 6b reveals that after treatment, the small pores in the rock cuttings are filled with a significant number of nano-particles, forming a dense plugging layer. The shale surface becomes smoother, and in areas with prominent fractures, the nano-plugging agents are densely adsorbed on the micro-cracks, effectively sealing the shale’s micro-fractures. This observation demonstrates the excellent plugging performance of the nano-plugging agent. The presence of cationic monomers in NPA enhances its adsorption onto the shale surface, allowing the nano-particles to efficiently fill and seal micro-cracks and micro-pores. Consequently, this reduces pressure transmission and prevents the intrusion of drilling fluid. These effects are beneficial for improving borehole stability during drilling operations.

### 3.7. Evaluation of Temperature-Resistant Stability Performance

**Figure 7:**
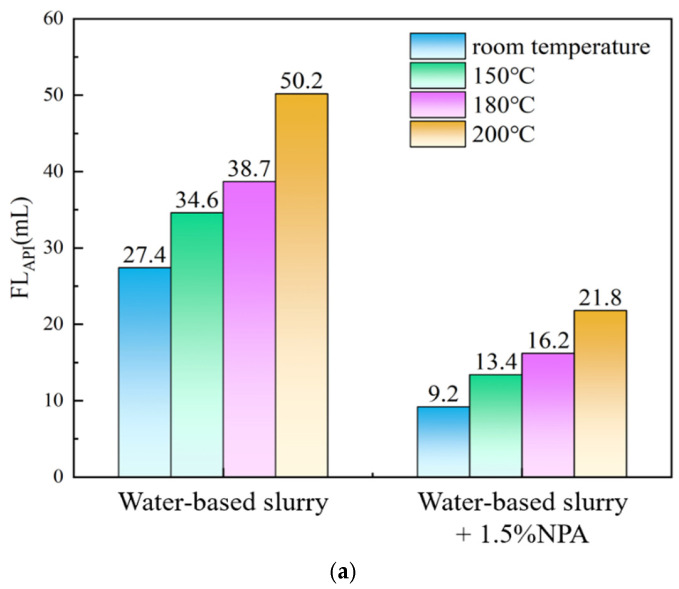

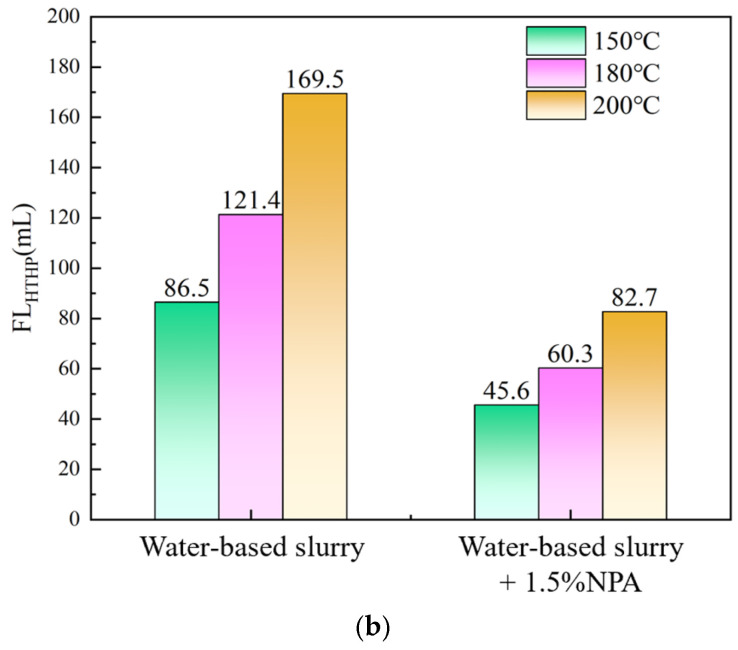
API filtration loss (**a**) and HTHP filtration loss (**b**) of drilling fluid base slurry at different aging temperatures.

Using the testing method outlined in Section 2.4.5, 1.5% nano-plugging agent (NPA) was added to the base slurry, and the changes in the drilling fluid’s rheological and filtration properties before and after aging at various temperatures are summarized in Table 4 and Figure 7. Table 4 shows that after aging at room temperature and elevated temperatures (150 °C, 180 °C, and 200 °C), the viscosity of the drilling fluid with 1.5% NPA decreases slightly. However, this change is minimal, indicating that the addition of NPA has little impact on the rheological properties of the drilling fluid. Figure 7 illustrates that as the temperature increases, the filtration loss of the drilling fluid also gradually rises. Nevertheless, compared to the base slurry at the same temperature, the drilling fluid containing 1.5% NPA exhibits significantly lower filtration loss. For instance, after aging at 180 °C, the API filtration loss is reduced from 38.7 mL to 16.2 mL, and the HTHP filtration loss is reduced from 121.4 mL to 60.3 mL, with the reduction rates of 58.1% and 50.3%, respectively. Even at 200 °C, the NPA maintains its ability to reduce filtration loss, effectively forming a plug within the drilling fluid. This superior performance can be attributed to the structural features of the NPA. The presence of benzene rings and sulfonic acid groups imparts strong rigidity and thermal stability to the molecular chain, minimizing decomposition under high-temperature conditions. Additionally, the cationic groups in NPA enhance its adsorption capability, allowing it to maintain excellent plugging performance even under extreme conditions.

### 3.8. Comparative Evaluation of Different Plugging Agents

**Figure 8:**
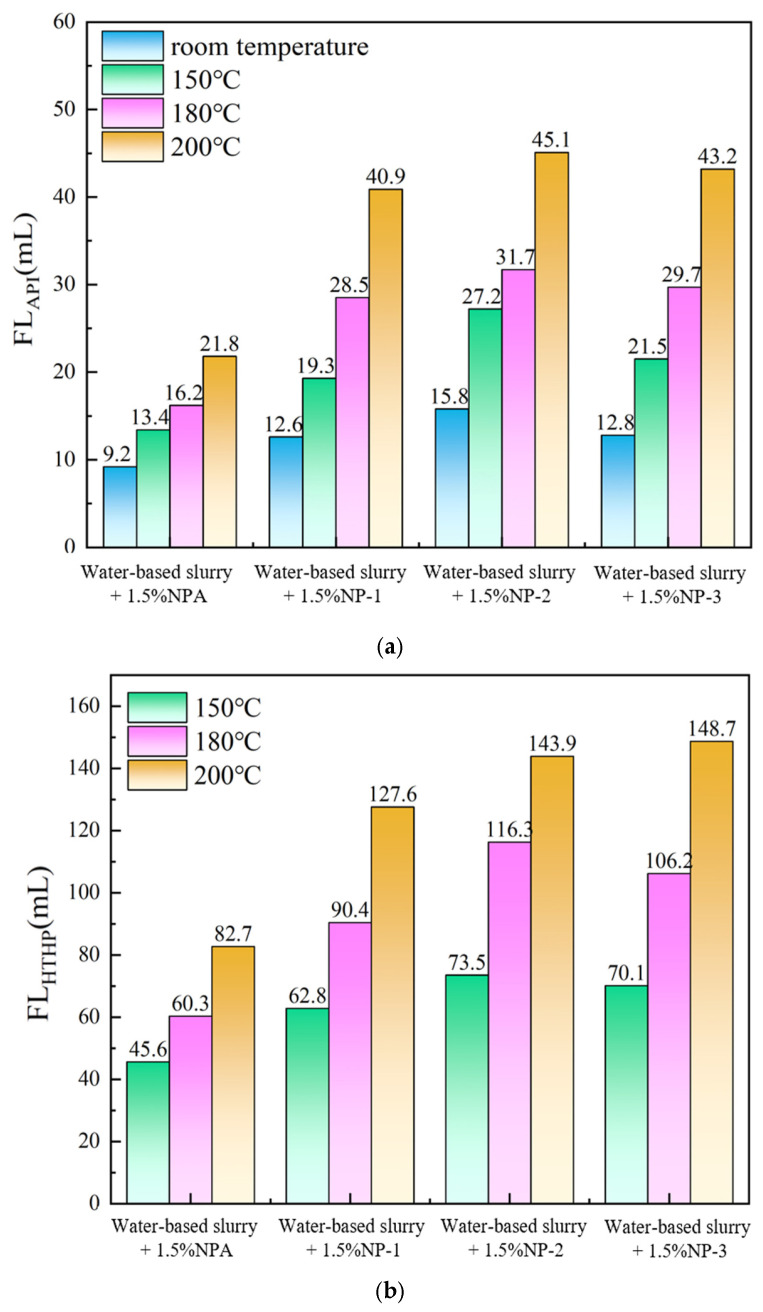
Comparative evaluation of the filtration loss of different plugging agents in drilling fluid base slurry. (**a**) API filtration loss for different plugging agents. (**b**) HTHP filtration loss of different plugging agents.

Using the testing method described in Section 2.4.6, a comparison of the rheological and filtration properties of different plugging agents in the drilling fluid base slurry is presented in Table 5 and Figure 8. Among them, NP-1 and NP-2 are nano-plugging agents, and NP-3 is a bituminous plugging agent. As shown in Table 5, after aging at room temperature and a high temperature of 150 °C, the AV and PV of drilling fluid base slurry with 1.5% nano-plugging agent are close to each other, and the difference is small. After aging at a high temperature of 200 °C, the AV and PV of drilling fluid with nano-plugging agent NP-1 decreased, and the decrease was larger, and the AV and PV of drilling fluid with NP-2 decreased first and then increased, which may be due to the degradation of the molecular chain fracture at high temperature. While NP-3 bituminous plugging agent has a better plugging effect and a smaller reduction in filtration loss under 150 °C, but under 180 °C and 200 °C, the filtration loss decreases rapidly and the viscosity becomes smaller, which indicates that the NP-3 plugging agent has a poorer ability to resist high temperature. As can be seen from Figure 8, after aging at a high temperature of 200 °C, the API filtration loss of drilling fluid with NP-1 added increased from 21.8 mL to 40.9 m, respectively, and the HTHP filtration loss increased from 45.6 mL to 127.6 mL, respectively, while the effect of both NP-2 and NP-3 blocking agents was lower than that of NP-1, and they basically lost the plugging ability. In summary, the loss reduction performance of the nano-plugging agent NPA was better than that of the nano-plugging agents NP-1, NP-2, and the bituminous plugging agent NP-3, indicating that the nano-plugging agent NPA has good plugging performance and high temperature resistance.

### 3.9. Mechanism of Action Analysis

**Figure 9:**
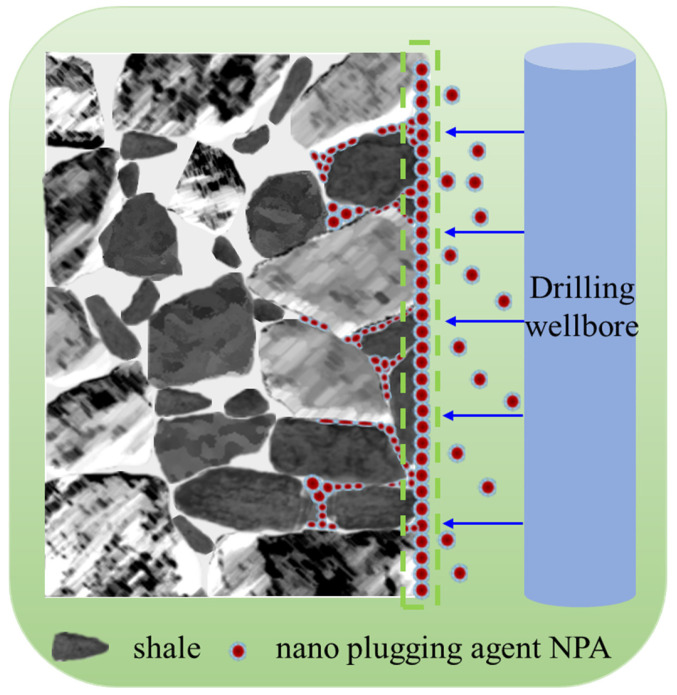
Schematic diagram of the mechanism of action of nano plugging agent NPA.

As illustrated in Figure 9, the mechanism of action of the nano-plugging agent NPA in the formation can be summarized as follows: The carboxyl group (-COOH) and sulfonic acid group (-SO_3_H) in NPA may form hydrogen bonding with the hydroxyl group (-OH) on the surface of clay minerals (e.g., kaolinite and montmorillonite) in shale to improve the adsorption capacity; the cationic monomer DMDAAC (cationic quaternary ammonium salt) contained in NPA may interact with the negatively charged silicate layer structure on the surface of shale to enhance the stability of the blocking; and particles may be tightly aligned through capillary action and physical adsorption to further reduce pore connectivity. The particles may be tightly arranged by capillary action and physical adsorption, further reducing the pore connectivity. Particle Size and Dispersion: The particle size of the nano-plugging agent is at the nanometer scale, significantly smaller than that of traditional plugging agents. This enables excellent dispersion in a water-based drilling fluids gel system, allowing the agent to penetrate tiny pores and cracks in the shale that conventional particles cannot reach, effectively addressing the limitations of traditional plugging agents. Deep Penetration into Pore Structures: The nano-plugging agent can infiltrate the micro-structures of pores and cracks, particularly in the transition area between the wellbore wall and the shale pores (depicted by the green dashed line in the figure). This capability results in deeper and more durable plugging effects, ensuring enhanced effectiveness over time. Surface Activity and High Surface Area: NPA exhibits outstanding surface activity and possesses a high specific surface area. Upon entering the shale, it tightly adheres to the pore walls. The particles accumulate on the shale surface and within the pores, forming a high-strength physical barrier. This barrier not only prevents water molecules from penetrating the shale but also inhibits the dissolution of substances that could chemically degrade the shale’s structure. Reducing Hydration and Expansion of Shale: Water in drilling fluids can cause shale to hydrate and expand, potentially leading to wellbore instability. The nano-plugging agent forms a physical barrier that reduces water diffusion and intrusion, thereby slowing shale expansion and minimizing the risk of wellbore collapse. Effective Plugging in Complex Structures: For shale formations with intricate fractures and micro-porous networks, the nano-plugging agent demonstrates the ability to penetrate hard-to-reach areas, providing more comprehensive and effective plugging. This makes it particularly well-suited for formations with complex pore structures. In summary, the nano-plugging agent NPA delivers superior plugging performance by filling microscopic pores and cracks, forming a robust physical barrier, and offering effective protection against hydration and chemical degradation of the shale.

## 4. Conclusions

This study successfully synthesized a nano-plugging agent (NPA) suitable for unconventional reservoirs. TEM observations revealed that NPA primarily exists as spherical or quasi-spherical particles and exhibits good dispersion in aqueous solutions. NPA demonstrates excellent high-temperature stability. At 150 °C, when 1.5% NPA was added to the base drilling fluid, the API and HTHP filtrate loss reductions were 66.4% and 47.3%, respectively. At 180 °C, the HTHP filtrate loss in the sand disk plugging test was reduced by 60.5%. Additionally, after NPA treatment, the specific surface area and pore volume of cuttings were 9.348 m^2^/g and 0.035 cm^3^/g, respectively, indicating that NPA formed an effective sealing layer on the shale surface and significantly enhanced the plugging effect. Further observations using scanning electron microscopy (SEM) confirmed that a large number of nanoparticles filled the micro-pores of the cuttings after plugging, forming a dense sealing barrier. Compared with other nano-plugging agents, NP-1 and NP-2, NPA exhibited the best filtrate loss reduction performance while maintaining the rheological properties of the base drilling fluid. NPA can penetrate micro-pore structures for deep plugging while also forming a physical barrier on the shale surface, preventing the infiltration of water molecules from the drilling fluid. This effectively inhibits shale hydration and swelling, ultimately achieving comprehensive plugging and wellbore stability. This study still has some limitations, mainly in the limited temperature range, the gap between the experimental conditions and the experimental conditions, etc. In the future, we can further optimize the synthesis process of NPA, evaluate the applicability of NPA in complex well conditions to improve its plugging efficiency and high-temperature stability, as well as explore the applicability of NPA in different geologic conditions to broaden the practical application of NPA in drilling fluids. Meanwhile, its applicability under different geological conditions was explored to broaden its practical application in drilling fluids.

## Figures and Tables

**Table 1:** Experimental materials and their functions.

Number	Designation	Function
1	Styrene (St)	Provides a benzene ring to enhance temperature resistance
2	2-acrylamido-2-methylpropanesulfonic acid (AMPS)	Polymerization reaction with acrylic acid, sulfonic acid group with temperature-resistant ability
3	Acrylic acid (AA)	Containing double bonds and carboxyl groups, chemically active
4	Dimethyldiallylammonium chloride (DMDAAC)	Containing double bonds and cationic quaternary ammonium groups, better hydrolytic stability and temperature and salt resistance
5	Potassium persulfate (KPS)	As an initiator
6	Silane coupling agent (KH570)	Modification of silicon dioxide nanoparticles
7	Span80	Co-surfactants
8	Anhydrous sodium carbonate (Na_2_CO_3_)	Adjusting the pH of the water-based drilling fluid base stock and softening the water
9	Anhydrous ethanol	Scrubbing synthesis product
10	Bentonite	Formulation of water-based drilling fluid slurries
11	Silicon dioxide nanoparticles (30 ± 5 nm)	Preparation of modified silica dioxide nanoparticles

**Table 2:** Effect of nano plugging agent NPA concentration on the rheological properties and filtration loss properties of drilling fluid base slurry.

NPA Concentration/wt%	Test Conditions	AV/(mPa·s)	PV/(mPa·s)	FL_API_/(mL)	FL_HPHT_/(mL)
0	BHR	12.5	8.5	27.4	-
	AHR	11.5	8.0	34.6	86.5
0.5	BHR	12.5	8.0	13.7	-
	AHR	11.0	7.0	17.2	63.1
1.0	BHR	13.5	7.5	10.5	-
	AHR	12.5	7.0	14.8	50.3
1.5	BHR	13.5	7.0	9.2	-
	AHR	12.0	7.0	13.4	45.6
2.0	BHR	14.0	7.5	8.6	-
	AHR	13.5	7.5	12.5	44.2

BHR: before hot rolling; AHR: after hot rolling.

**Table 3:** Specific surface area and pore analysis of shale in different treatment states.

Test Items	Original Shale	Deionized Water Treatment of Shale	NPA Treatment Shale
Specific surface area/(m^2^/g)	6.721	16.416	9.348
pore volume/(cm^3^/g)	0.016	0.047	0.035

**Table 4:** Nano plugging agent NPA temperature-resistant performance test.

Systems	Test Conditions	AV/(mPa·s)	PV/(mPa·s)	FL_API_/(mL)	FL_HPHT_/(mL)
Base slurry	room temperature	12.5	8.5	27.4	-
	150 °C/16 h	11.5	8.0	34.6	86.5
	180 °C/16 h	9.0	7.5	38.7	121.4
	200 °C/16 h	7.0	5.0	50.2	169.5
Base slurry + 1.5%NPA	room temperature	13.5	7.0	9.2	-
	150 °C/16 h	12.0	7.0	13.4	45.6
	180 °C/16 h	10.5	8.0	16.2	60.3
	200 °C/16 h	8.0	6.5	21.8	82.7

**Table 5:** Different plugging agent NPA temperature-resistant performance test.

Systems	Test Conditions	AV/(mPa·s)	PV/(mPa·s)	FL_API_/(mL)	FL_HPHT_/(mL)
Base slurry + 1.5%NPA	room temperature	13.5	7.0	9.2	-
	150 °C/16 h	12.0	7.0	13.4	45.6
	180 °C/16 h	10.5	8.0	16.2	60.3
	200 °C/16 h	8.0	6.5	21.8	82.7
Base slurry + 1.5%NP-1	room temperature	9.0	6.5	12.6	-
	150 °C/16 h	8.5	6.0	19.3	62.8
	180 °C/16 h	7.0	5.0	28.5	90.4
	200 °C/16 h	6.0	4.5	40.9	127.6
Base slurry + 1.5%NP-2	room temperature	12.5	6.0	15.8	-
	150 °C/16 h	9.0	4.5	27.2	73.5
	180 °C/16 h	8.5	5.5	31.7	116.3
	200 °C/16 h	10.5	7.0	45.1	143.9
Base slurry + 1.5%NP-3	room temperature	12.5	6.5	12.8	-
	150 °C/16 h	11.0	6.0	21.5	70.1
	180 °C/16 h	8.0	5.5	29.7	106.2
	200 °C/16 h	6.0	5.0	43.2	148.7

## Data Availability

The original contributions presented in this study are included in the article. Further inquiries can be directed to the corresponding author.

## Abbreviations

HTHP—high-temperature high-pressure; API—American Petroleum Institute; WBDFs—water-based drilling fluids; AMPS—2-acrylamido-2-methylpropanesulfonic acid; AM—acrylamide; St—styrene; KPS—potassium persulfate; AA—acrylic acid; DMDAAC—dimethyldiallylammonium chloride; KH570—silane coupling agent; AV—apparent viscosity; PV—plastic viscosity.
